# Gibberellic Acid-Induced Regulation of Antioxidant–Flavonoid Channels Provides Protection Against Oxidative Damage in Safflower Under Salinity Stress

**DOI:** 10.3390/plants15020267

**Published:** 2026-01-15

**Authors:** Zhiling Li, Xiaoyu Liu, Weijie Meng, Julong Shangguan, Jian Zhang, Imran Ali, Na Yao, Min Zhang, Naveed Ahmad, Xiuming Liu

**Affiliations:** 1 College of Life Sciences, Engineering Research Center of the Chinese Ministry of Education for Bioreactor and Pharmaceutical Development, Jilin Agricultural University, Changchun 130118, China; 2 Institute for Safflower Industry Research of Shihezi University/Pharmacy College of Shihezi University/Key Laboratory of Xinjiang Phytomedicine Resource and Utilization, Ministry of Education, Shihezi 832003, China; 3 Department of Botany, Kohat University of Science and Technology, Kohat 26000, Khyber Pakhtunkhwa, Pakistan; 4 Monitoring and Testing Center for Ginseng and Antler Products, Ministry of Agriculture and Rural Affairs, Jilin Agriculture University, Changchun 130118, China

**Keywords:** gibberellin, salt stress, redox homeostasis, gene expression, hydroxy safflor yellow A, *Carthamus tinctorius* L.

## Abstract

Salinity is a major constraint that compromises safflower performance by disrupting redox balance and metabolic homeostasis. Although hormonal mechanisms for improving plant resilience to abiotic stresses have been reported, the mechanistic role of gibberellic acid (GA_3_)-induced regulation of safflower tolerance to salinity remains unclear. This study aimed to investigate the impact of exogenous GA_3_ application under normal and saline conditions to evaluate its effects on growth, physiology, redox regulation, and flavonoid biosynthesis in safflower. Using phenotypic, physiological, biochemical, and gene expression analysis, it is suggested that GA_3_ significantly alleviates salt stress by integrating antioxidant defense and flavonoid biosynthesis. The results of phenotypic and physiological assessments showed that GA_3_ at 400 mg/L GA_3_ in safflower seedlings suggests enhanced vegetative growth and photosynthetic performance. Under salt stress, GA_3_ significantly alleviated oxidative damage by reducing H_2_O_2_, $O_{2}^{-}$, and malondialdehyde (MDA) levels, while enhancing osmoprotective compounds such as proline, soluble sugars, proteins, and chlorophyll. GA_3_ also significantly increased the activity of antioxidant enzymes (SOD, POD, CAT, APX, GST, DHAR, and Prx), accompanied by the transcriptional upregulation of their corresponding genes, indicating GA_3_-mediated regulation of redox homeostasis at both biochemical and molecular levels. In parallel, GA_3_ enhanced the accumulation of major flavonoids, particularly hydroxy safflor yellow A (HSYA), with strong induction of key HSYA biosynthetic genes (*CtF6H*, *CtCGT*, *Ct2OGD1*), whereas salinity alone suppressed their expression. In contrast, the quercetin branch displayed a regulatory bottleneck at *CtF3H*, which remained suppressed under all treatments, although upstream genes were GA_3_-responsive. Together, these findings demonstrate that GA_3_ enhances salinity tolerance in safflower by simultaneously activating antioxidant defenses and stimulating flavonoid biosynthesis, providing mechanistic insight with practical implications for developing salt-resilient safflower varieties.

## 1. Introduction

Salinity stress is one of the most severe environmental challenges that adversely affects agricultural productivity globally [1]. It is estimated that salt stress alone affects more than 20–33% of cultivated and irrigated land [2]. These detrimental effects are continuously expanding due to climate change, poor irrigation practices, and soil degradation [3]. The increasing salinization of arable land requires the development of effective strategies to enhance the salt tolerance capacity of essential crops. Safflower (*Carthamus tinctorius* L.) has garnered attention as an important oilseed crop due to its adaptability to arid and semi-arid environments [4,5,6]. However, like many other crops, safflower cultivation and yield are often compromised by salt stress, leading to inhibited growth and reduced yield [7,8]. While conventional breeding focuses on inherent tolerance, innovative phytoprotectant strategies that modulate key physiological and molecular pathways present a promising alternative for rapidly enhancing crop resilience. This study provides the first systematic stress alleviation strategy by utilizing the potential of the exogenous application of gibberellic acid (GA_3_) to specifically prime the antioxidant defense and flavonoid biosynthesis pathways, critical channels for mitigating oxidative damage in safflower under salinity conditions.

Recent advances in plant physiology have highlighted the effective use of phytohormones as growth regulators in modulating salt stress tolerance pathways [9,10,11,12]. Among these, GA_3_, a group of diterpenoid plant hormones, has emerged as a key regulator with multifaceted roles in enhancing crop yield and production [13,14]. The exogenous application of GA_3_ is a promising recent approach for the modulation of plant growth and the development and the enhancement of resilience against abiotic stresses [15,16,17]. For instance, GA_3_ treatments have been shown to modulate various processes such as seed germination [18], root development [19], shoot and stem elongation [20], flower development [21], and fruit development [22]. In addition, the application of exogenous GA_3_ has been demonstrated to alleviate the adverse effects of abiotic stresses, including salinity stress [23,24,25], and it has been shown to enhance the metabolic activity of defense-related secondary metabolites such as flavonoid biosynthesis [26,27].

Under stress conditions, plants undergo profound adverse effects, owing to disrupted cellular osmotic balance, impaired nutrient uptake, and reduced photosynthetic efficiency [28,29]. Elevated ROS levels are common during salt stress, leading to lipid peroxidation and cell membrane damage, indicated by increased malondialdehyde (MDA) levels [30,31]. A wide range of studies have suggested that GA_3_ application enhances chlorophyll biosynthesis, improves stomatal conductance, and maintains photosynthetic performance under both optimal and stress conditions [27,32,33]. It effectively provides protection against oxidative damage by regulating ROS production and activating enzymatic and non-enzymatic antioxidant systems [34,35]. The foliar application of GA_3_ has been reported to enhance the activity of antioxidant enzymes such as superoxide dismutase (SOD), catalase (CAT), ascorbate peroxidase (APX), and glutathione reductase (GR) by increasing pools of low-molecular-weight antioxidants [36]. Similarly, GA_3_ can alter the expression of genes encoding these enzymes and interact with DELLA regulators that cross-talk with ROS-responsive transcriptional networks, thereby coordinating transcriptional and post-translational responses that restore redox balance under stress [37].

Flavonoids are biologically active metabolites widely recognized for their redox-based antioxidant properties, which contribute significantly to the alleviation of abiotic stress in plants [38,39]. Recent insights indicated that GA_3_ also acts as effective elicitor that modulates secondary metabolite production. For example, several studies have demonstrated that GA_3_ promotes flavonoid biosynthesis by modulating the transcriptional and enzymatic activities involved in phenolic metabolism [26,40,41,42]. These bioactive metabolites not only contribute to ROS scavenging, but also act as signaling molecules that regulate broader stress-responsive networks [43,44,45]. It has been shown that the overexpression of anthocyanidin synthase in *Oryza sativa* L. leads to an accumulated complex of flavonoids with enhanced antioxidant properties [46]. Similarly, the modification of the flavonoid biosynthetic pathway in *Solanum lycopersicum* L. suggested improved antioxidant levels [47]. Despite these promising findings, the underlying physio-chemical and molecular mechanisms by which GA_3_ orchestrates salt-tolerant responses by regulating antioxidant–flavonoid channels in safflower remain poorly understood.

The present study investigates the impact of GA_3_ on safflower growth, photosynthetic performance, oxidative stress markers, antioxidant enzyme activity, and flavonoid accumulation under normal and saline conditions. Furthermore, by profiling the expression of key genes associated with antioxidative and flavonoid biosynthesis pathways, this work aims to unravel the molecular frameworks underpinning GA_3_-mediated salinity tolerance. Understanding these interconnected regulatory networks will not only advance current knowledge of hormonal-induced stress adaptation, but also provide a foundation for developing GA_3_-based strategies to enhance safflower resilience and productivity under saline environments.

## 2. Results

### 2.1. Gibberellic Acid Induced Significant Phenotypic and Photosynthetic Changes in Safflower

**Figure 1:**
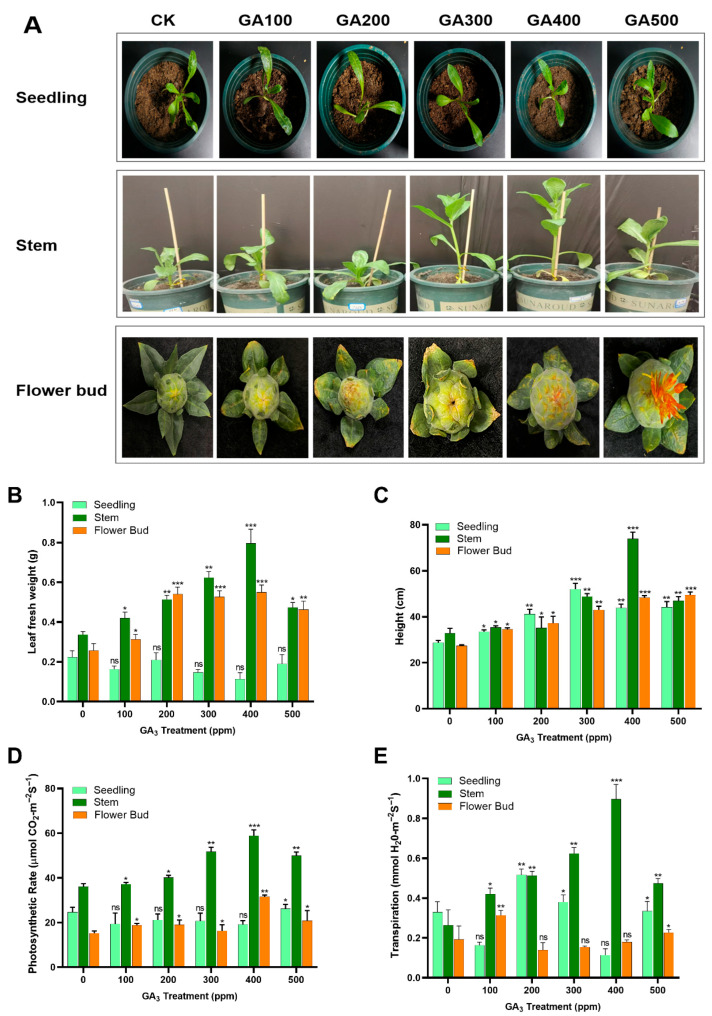
Effects of various gibberellic acid concentrations on safflower at different growth stages. (**A**) Phenotypic variations at seedling, stem, and flower bud stages treated with different GA_3_ concentrations. (**B**,**C**) Measurement of leaf fresh weight and plant height (**D**,**E**) Quantification of photosynthetic and transpiration rate. Data are presented as mean ± SD, n  =  3 biological replicates. The asterisks indicate statistically significant differences as compared to the control group (* *p* ≤ 0.05, ** *p* ≤ 0.01, *** *p* ≤ 0.001).

The present study first screened the effect of different gibberellic acid concentrations (0, GA100, GA200, GA300, GA400, GA500) on safflower growth and other key physiological traits in seedling, stem, and flower bud tissues under normal conditions (Figure S1). GA_3_ treatments showed no obvious phenotypic changes at the seedling stage, whereas the flower bud stage treated with GA400 and GA500 exhibited early flowering when compared to the control group (Figure 1A). Noticeably, at the stem stage, significant growth-promoting effects were observed when safflower plants were treated with GA300 and GA400, respectively (Figure 1A). Similarly, the leaf biomass and plant height were also investigated under various concentrations of GA_3_. The results of the leaf biomass quantification showed that stem tissue showed a more significant response than seedlings and flower bud tissues. Treatment with GA400 obtained the highest leaf biomass in stem tissues, reaching its maximum when compared to the control group (Figure 1B). The plant height indicators also reached to their maximum (72.03 ± 1.46 cm) in the stem tissue when treated with GA400, approximately three-time times greater than that of the control group (Figure 1C). Furthermore, the rate of photosynthesis and transpiration after GA_3_ treatment were found to be significantly higher in the stem tissues, reaching their peak at GA400 concentration than those the control, respectively (Figure 1D,E). The mean values of photosynthetic and transpiration rate in the stem tissues in the GA400 treatment group were 39% and 67% higher than those in the control group, respectively. Based on these findings, we conclude that GA400 is the optimal GA_3_ concentration, with the stem tissue exhibiting enhanced safflower growth and physiological performance.

### 2.2. Gibberellic Acid Mitigates Salinity-Induced Oxidative Damage by Regulating ROS Contents and Osmoprotectant Accumulation

**Figure 2:**
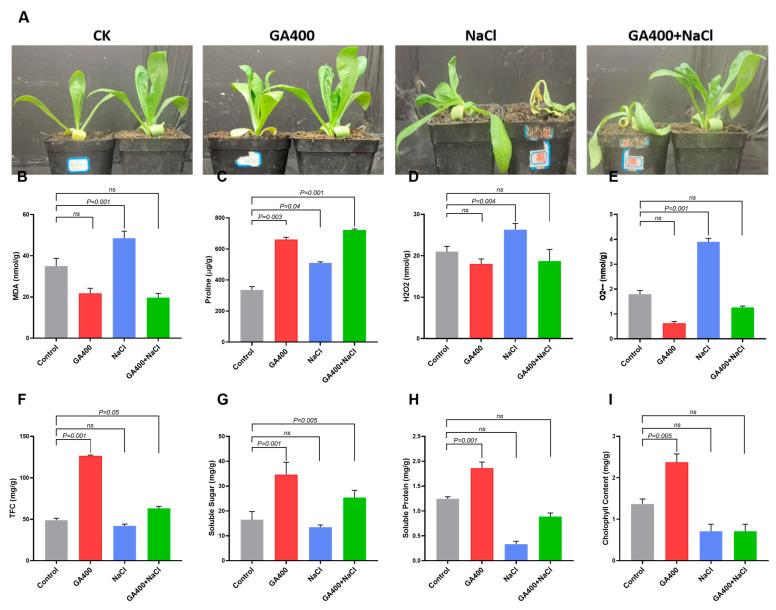
GA_3_-induced biochemical and physiological effects in safflower leaves under salt stress. (**A**) Phenotypic variation of the control. (**B**) MDA content. (**C**) Proline content. (**D**) Hydrogen peroxide content. (**E**) Superoxide anion ( $O_{2}^{-}$) content. (**F**) Total flavonoid content. (**G**) Soluble sugar content. (**H**) Soluble protein content. (**I**) Chlorophyll content. Data are presented as mean ± SD; n  =  3 biological replicates.

To determine how GA_3_ influences safflower tolerance to oxidative damage under salinity stress, key biochemical and physiological parameters under four experimental conditions were investigated. Results showed that the MDA and reactive oxygen species (ROS) content (H_2_O_2_ and $O_{2}^{-}$) were higher under salinity stress than the control and other treatment groups (Figure 2A–D). Salt stress triggers excessive ROS production, causing elevated MDA, a key indicator of oxidative damage. On the other hand, GA_3_ treatment alone and combined GA_3_ + NaCl stress demonstrated a significant reduction in MDA and ROS content (H_2_O_2_ and $O_{2}^{-}$), when compared to the control group (Figure 2A–D). According to the results of proline quantification, it was observed that GA_3_ and combined GA_3_ + NaCl treatment led to a significant increase in the proline content compared to the control group (Figure 2E).

Similarly, salinity stress alone also leads to higher accumulation of proline content than the control, but a lower amount than GA_3_ alone and combined GA_3_ + NaCl. Similarly, total flavonoid content showed a distinct treatment-dependent variation, with the highest accumulation observed in plants treated with GA_3_ alone, followed by the combined GA_3_ + NaCl stress treatment (Figure 2F). Plants exposed to salinity stress alone exhibited reduced flavonoid levels compared to the control group (Figure 2F). A comparable pattern was observed for soluble sugar content, where GA_3_ application alone significantly enhanced sugar accumulation relative to all other treatments. The combined GA_3_ + salt treatment also showed elevated levels, though slightly lower than GA_3_ alone, while salinity stress alone resulted in a decrease compared to the control (Figure 2G). Soluble protein and chlorophyll content followed the same trend, with GA_3_ treatment alone inducing the highest increase, followed by the combined treatment. In contrast, salinity stress alone caused a reduction in protein content compared to the control plants (Figure 2H,I).

### 2.3. GA_3_ Significantly Enhances Antioxidant Enzymatic Networks to Strengthen Redox Homeostasis Under Salinity Stress

**Figure 3:**
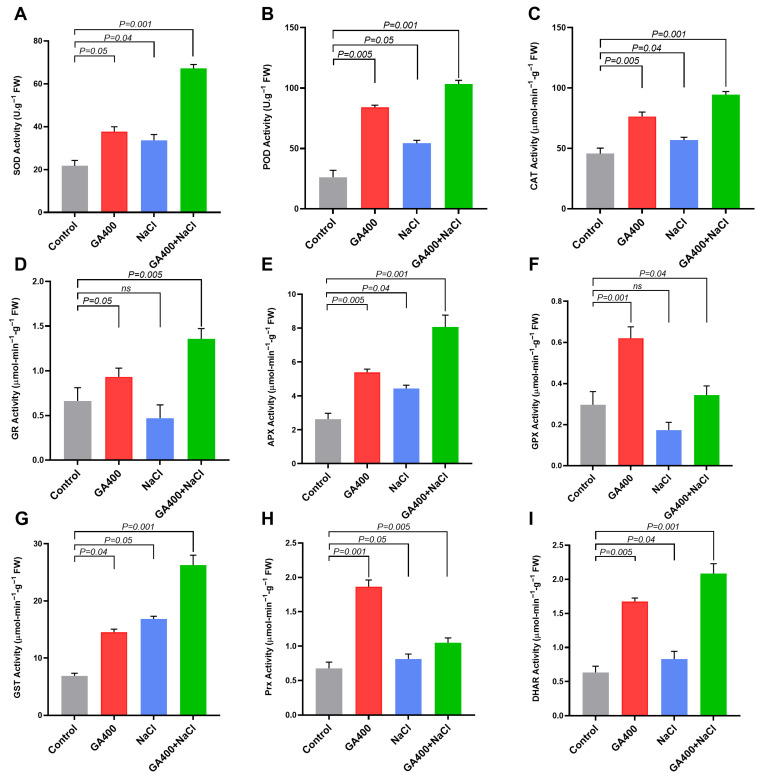
Effects of GA_3_ on the activity of different antioxidant enzymes in safflower under salinity stress. Activity of major antioxidant enzymes in safflower leaves under four treatments: (**A**) superoxide dismutase (SOD), (**B**) peroxidase (POD), (**C**) catalase (CAT), (**D**) glutathione reductase (GR), (**E**) ascorbate peroxidase (APX), (**F**) glutathione peroxidase (GPX), (**G**) glutathione-S-transferase (GST), (**H**) peroxiredoxin (Prx), and (**I**) dehydroascorbate reductase (DHAR). Data are presented as mean ± SD (n = 3).

To further understand how GA_3_ application influences the enzymatic antioxidant defense system in safflower seedlings treated with NaCl stress, the activity of superoxide dismutase, peroxidase, catalase, glutathione reductase, ascorbate peroxidase, glutathione peroxidase, glutathione-S-transferase, peroxiredoxin, and dehydroascorbate reductase using four treatment groups were investigated. Compared to the untreated control samples, the activity of most antioxidant enzymes, such as SOD, POD, CAT, APX, and DHAR, increased significantly (*p* ≤ 0.001) in the combined GA_3_ + NaCl stress treatment (Figure 3). Similarly, GA_3_ and NaCl stress alone also triggered a significant increase in the activity of SOD, POD, and CAT when compared with the untreated control group (Figure 3). In contrast, enzymes such as GPX and Prx exhibited significantly higher activity (*p* ≤ 0.001) than the control treatment group when the plants were treated with GA_3_ alone, followed by the combined GA_3_ + NaCl stress treatment. The activity of these enzymes demonstrated a contrasting pattern under NaCl stress alone, with a slight increase in Prx activity, whereas GR activity was significantly reduced when compared to the untreated control group (Figure 3).

Moreover, the trend of a significant increase in the activity of the GST and DHAR enzymes was found in the combined GA_3_ + NaCl stress treatment group (*p* ≤ 0.001) compared to the control. However, NaCl stress also induces a moderate increase in GST activity compared to GA_3_ alone, but a significant increase (*p* ≤ 0.05) compared to the untreated control group, whereas DHAR activity was comparatively higher (*p* ≤ 0.005) than that of the NaCl stress and the untreated control group (Figure 3). These results suggest that GA_3_ could have potential inherent efficient-scavenging properties by amplifying enzymatic antioxidant channels, thereby facilitating efficient redox homeostasis in salinity-stressed safflower.

### 2.4. Transcriptional Activation of Key Antioxidant Pathway Genes by GA_3_ Under Salinity Stress

**Figure 4:**
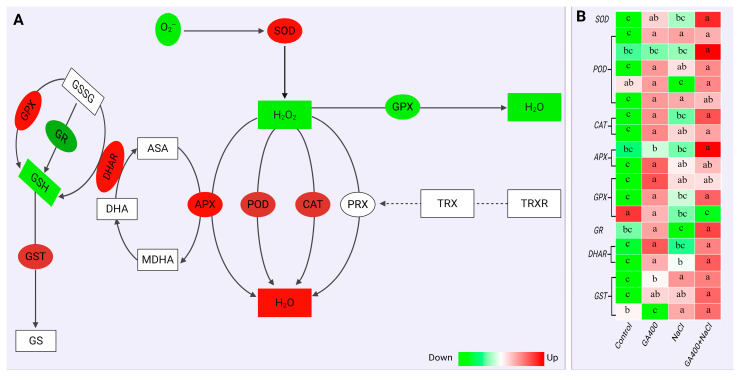
GA3-induced expression profiling of antioxidant pathway genes in safflower under salinity stress. (**A**) Schematic overview of the enzymatic antioxidant network showing interactions among SOD, CAT, POD, APX, GPX, GR, DHAR, GST, and components of the ascorbate–glutathione cycle. Upregulated genes or enzymes are indicated in red, while downregulated components appear in green. (**B**) Heatmap showing relative expression levels of antioxidant-related genes under four treatments: control (CK), GA_3_ alone (400 ppm), NaCl alone (100 mM), and combined GA_3_ + NaCl. treatment. Expression values are normalized to internal controls and presented as mean fold change. The letters (a, b, c, ab, and bc) are means ± SD (n = 3).

To elucidate the molecular basis of GA_3_-mediated enhancement of safflower salt tolerance via the regulation of antioxidant enzymatic pathways, real-time quantitative PCR (qRT-PCR) was employed to quantify the transcriptional responses of key antioxidant pathway genes under four treatment groups including the untreated control, GA_3_ treatment, NaCl stress, and combined GA_3_ + NaCl treatment. The expression levels of one *SOD-*, five *POD-*, two *CAT-*, one *APX-*, one *GPX-*, one *GR-*, two *DHAR-*, and three *GST*-encoding gene(s) were significantly upregulated (*p* ≤ 0.001) under the combined GA_3_ + NaCl treatment, whereas one *GPX* gene was downregulated relative to the untreated control group (Figure 4). Under GA3 treatment alone, the expression levels of four *POD*, two *CAT*, one *APX*, three *GPX*, one GR, two *DHAR*, and two GST gene(s) were significantly upregulated (*p* ≤ 0.001). Under the same GA_3_ treatment, except for one GST that was significantly downregulated, one *POD*, one *GST*, one *APX,* and one *SOD* gene did not change significantly when compared to the untreated control group (Figure 4). Furthermore, NaCl stress alone also induced changes in the transcription level of two *POD* and two *GST* genes, which showed significant upregulation (*p* ≤ 0.001), whereas one *POD*, one *CAT*, one *APX*, one *GPX*, and one *GST* gene showed a slight increase in their expression compared to the untreated control group (Figure 4). Except for one *GR* and one *POD* gene, which showed significant downregulation, the expression level of one *SOD*, one *POD*, one *CAT*, one *APX*, two *GPX,* and one *DHAR* gene(s) did not change significantly under NaCl stress when compared to the untreated control group. These findings suggested that the transcriptional profiles of antioxidant-related genes aligned closely with the activity trends of their respective antioxidant enzymes, indicating that GA_3_ enhances safflower tolerance to salt stress by modulating key enzymatic antioxidant pathways at the transcriptional level.

### 2.5. GA_3_ Enhances Flavonoid Accumulation by Upregulating HSYA Biosynthetic Gene Expression Under Salinity Stress

**Figure 5:**
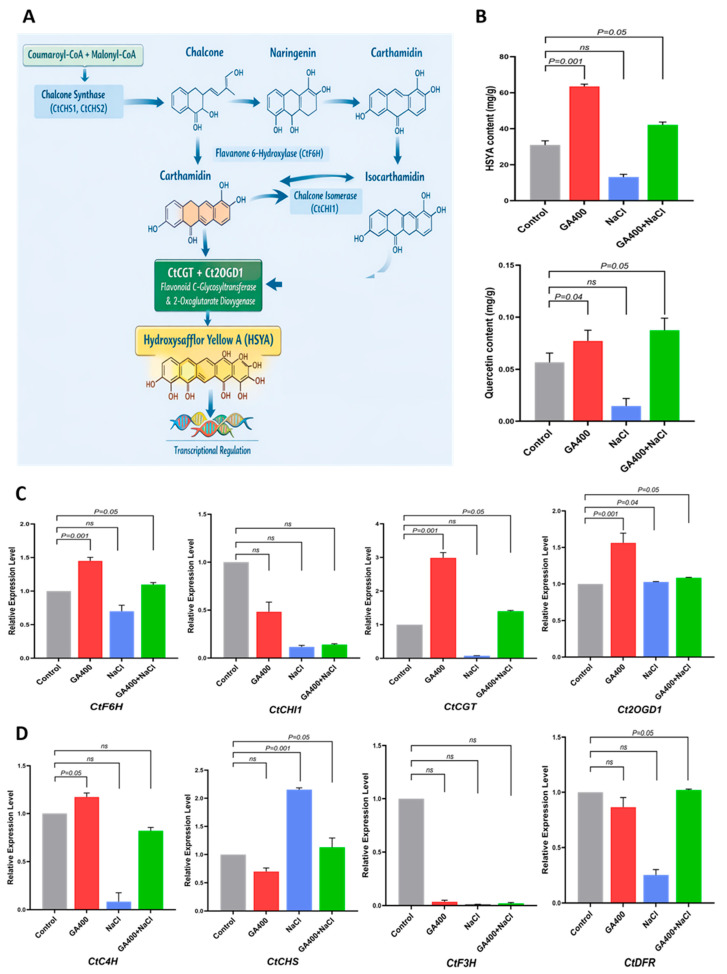
Effect of GA_3_ on accumulation of flavonoids and their biosynthetic gene expression in safflower under salinity stress. The proposed pathway of HSYA biosynthesis in safflower (**A**); HSYA and quercetin content (**B**); relative expression of biosynthetic genes involved in the HSYA biosynthesis pathway (**C**) and the quercetin biosynthesis pathway (**D**). Control (CK), GA_3_ (400 ppm), NaCl (100 mM), and GA_3_ + NaCl treatments. Data are presented as mean ± SD (n = 3). *Cinnamate 4-hydroxylase* (*CtC4H*), *chalcone synthase* (*CtCHS*), *flavonoid-3-hydroxylase* (*CtF3H*), *flavonoid-6-hydroxylase* (*CtF6H*), *chalcone isomerase* (*CtCHI1*), *flavonoid di-C-glycosyltransferase* (CtCGT), *dihydroflavonol 4-reductase* (*CtDFR*), and *2-oxoglutarate-dependent dioxygenase* (*Ct2OGD1*).

To determine whether GA_3_ application also contributes to flavonoid-mediated mitigation of salinity stress in safflower, the quantification of hydroxy safflor yellow A (HSYA) and quercetin levels was performed. Simultaneously, the expression levels of key genes associated with HSYA and quercetin biosynthetic pathways were also investigated. As shown in Figure 5A, GA_3_ application significantly enhanced HSYA accumulation (*p* ≤ 0.001) and a moderate but statistically significant increase (*p* ≤ 0.05) was also observed under the combined GA_3_ + NaCl treatment compared with both the control and salt-only treatments. Conversely, NaCl stress led to a significant reduction in HSYA content relative to all other groups, indicating that GA_3_ can alleviate salt-induced suppression of HSYA production. A similar trend was observed for quercetin levels; however, the most substantial increase occurred in the GA_3_ + salt treatment group, followed by GA_3_ treatment alone (Figure 5B). In contrast, quercetin content significantly declined under salt stress, consistent with the pattern observed for HSYA (Figure 5B).

For the expression analysis, a total of four genes (known to regulate the safflower HSYA biosynthetic pathway) [48] were selected to investigate GA_3_-induced expression bias under NaCl stress. As shown in Figure 5, compared to the untreated control group, except for *chalcone isomerase (CtCHI1)*, which showed downregulation, GA_3_ treatment induced significant upregulation (*p* ≤ 0.001) of *flavonoid-6-hydroxylase* (*CtF6H*), *flavonoid di-C-glycosyltransferase (CtCGT)*, and *2-oxoglutarate-dependent dioxygenase (Ct2OGD1)*, followed by the combined GA_3_ + salt treatment group. On the contrary, salt treatment alone induced significant downregulation of *CtF6H*, *CtCHI*, and *CtCGT*, except for *Ct2OGD1*, which showed slight upregulation than that of the control treatment (Figure 5C). Similarly, four key structural genes (known to regulate the quercetin biosynthetic pathway) [49] were also selected to observe their GA_3_-induced expression. As demonstrated in Figure 5D, the expression level of *Cinnamate 4-hydroxylase (CtC4H)* was slightly upregulated under GA_3_ treatment alone; however, it was significantly downregulated under salt and GA_3_ + NaCl treatment than that of the control group (Figure 5D). The expression of *chalcone synthase (CtCHS)* showed significant upregulation (*p* ≤ 0.001) when subjected to salt treatment alone and was slightly increased under GA_3_ + NaCl treatment when compared with the untreated control. Noticeably, the expression level of *flavonoid-3-hydroxylase (CtF3H)* showed significant downregulation, whereas the expression of *dihydroflavonol 4-reductase* (*CtDFR)* did not change significantly in any of the three treatment groups compared to the untreated control (Figure 5D). Taken together, these results demonstrate that HSYA accumulation and the expression of its biosynthetic genes are significantly correlated under both GA_3_ alone and GA_3_ + NaCl treatments and this was more obvious than in quercetin. This highlighted the pivotal role of GA_3_ in sustaining HSYA biosynthesis, particularly through transcriptional regulation, which appears to be a key mechanism contributing to enhanced salt stress tolerance in safflower.

## 3. Discussion

### 3.1. Salinity Stress Is a Severe Threat to Safflower Growth

Widespread salinity conditions greatly influence terrestrial ecosystems and increasingly affect agricultural land where crops such as safflower are cultivated [50]. Previously, it has been demonstrated that salt stress induced a significant reduction in leaf growth [51] and the fresh weight of safflower [7]. The study also showed that salinity stress poses negative effects on ion composition, including lipid and fatty acid content in safflower [52]. Another study demonstrated the detrimental effects of salinity on proline accumulation, antioxidant enzyme activity, and ion content (Na^+^, K^+^, and Ca^2+^) in different safflower cultivars [8]. Similarly, several physiological and biochemical processes were inhibited when safflower seedlings of cv. Goldasht were treated with salinity stress [53]. In the present study, NaCl stress treatment increased the MDA and ROS content (H_2_O_2_ and $O_{2}^{-}$) in safflower seedlings (Figure 2). In accordance with these results, several prior studies reported a consistent rise in MDA and hydrogen peroxide content under NaCl stress, reflecting severe membrane injury in stressed plants [54,55,56]. It was also observed in this study that NaCl stress impedes healthy biochemical composition (TFC, soluble sugar, soluble protein, and chlorophyll content), but increases proline content. Antioxidant enzyme activity (SOD, POD, CAT, APX, GST) was also enhanced, whereas the activities of GR and GPX were significantly reduced compared to the control (Figure 3). Comparable findings were observed in other studies that reported increased antioxidant enzyme activity in *Zea mays* [57] and *Mentha pulegium* [57] under salt stress. Our results also demonstrated that salt stress leads to the downregulation of key biosynthetic genes involved in antioxidant, HSYA, and quercetin pathways, indicating that salt stress inflicts severe damage to the safflower plants at a transcriptional level (Figure 4 and Figure 5). Consistent with these findings, studies in *Arachis hypogaea* [54], *Ginkgo biloba* [55], and *Cyclocarya paliurus* [56] revealed that salt stress significantly enhanced the transcription of genes participating in flavonoid and antioxidant biosynthetic pathways.

### 3.2. Gibberellic Acid Activates Redox Homeostasis in Safflower Under Salinity Stress

As an essential plant growth regulator, GA_3_ plays a crucial role in coordinating growth and developmental processes and improving stress tolerance under diverse abiotic conditions [58,59]. Studies on *Triticum aestivum* [60] and *Sorghum bicolor* [61] demonstrated that GA_3_ can effectively overcome the negative effects of salt stress. Similarly, treatment with GA_3_ has been demonstrated to enhance antioxidant enzyme activity and improve the growth performance, germination, and yield quality of a variety of crops under salinity stress [62,63]. However, GA_3_ application is subject to dose-dependent and tissue-specific manners in order to regulate plant growth [64,65]. In this study, different concentrations of GA_3_ treatment exerted distinct, tissue-specific and dose-dependent effects on safflower growth, with the stem indicating the most pronounced response across all measured traits at the GA400 dose (Figure 1). The significant responsiveness of stem tissues observed in this study is consistent with the known role of GA in regulating internode elongation and stem vascular differentiation [66]. Stems possess higher GA sensitivity due to elevated expression of GA receptors and downstream signaling components, enabling stronger physiological responses compared to seedlings or reproductive tissues [67]. Similarly, enhanced photosynthetic and transpiration rates at GA400 further suggest that optimal GA_3_ dosage improves stomatal conductance, chlorophyll biosynthesis, and CO_2_ assimilation capacity, thereby supporting increased biomass production [68]. In contrast, lower GA_3_ concentrations (GA100–GA200) were likely insufficient to fully activate GA signaling pathways, resulting in limited physiological responses. Conversely, higher concentrations (GA500), although capable of inducing early flowering at the bud stage, did not further enhance vegetative growth, indicating a threshold beyond which GA_3_ no longer promotes vegetative performance and may redirect assimilates toward reproductive development. Studies on horticultural crops such as petunia and lettuce have shown that optimal intermediate GA_3_ concentrations significantly enhance plant height, biomass, and yield, but higher doses can negatively impact morphological traits and fresh weight accumulation, indicating that a concentration threshold exists, beyond which additional hormone is not beneficial [69,70].

Our findings further demonstrated that GA_3_ significantly alleviated NaCl-induced oxidative damage in safflower by reducing ROS content (H_2_O_2_ and O_2_^−^) and MDA levels compared with plants under salt stress alone (Figure 2). GA_3_ (alone and in combination with NaCl) enhanced proline, flavonoids, soluble sugars, proteins, and chlorophyll, indicating that GA_3_ could effectively mitigate salt-induced damage, likely by modulating stomatal regulation, promoting metabolite and osmoprotectant synthesis, and enhancing antioxidant defense (Figure 2). This study also found that GA_3_ noticeably enhanced the enzymatic antioxidant defense system of safflower under NaCl stress, with combined GA_3_ + NaCl treatment producing the highest activity of major ROS-scavenging enzymes (SOD, POD, CAT, APX, GST, DHAR). GA_3_ alone strongly promoted GPX and Prx activity, while NaCl stress alone showed weaker or inconsistent induction and even suppressed GR activity (Figure 3). Consistently, in linseed (*Linum usitatissimum*) and maize crops, exogenous GA_3_ application under salinity enhanced the activity of key antioxidant enzymes, including CAT, SOD, and POD, supporting a GA_3_-mediated reinforcement of redox defense [65,71]. Similar reports have demonstrated the activity of the above-mentioned enzymes in other plants under salinity and GA_3_ treatments [72,73]. Collectively, these results suggest that GA_3_ improve redox homeostasis by amplifying multiple antioxidant enzymatic channels, leading to enhanced safflower tolerance to salt-induced oxidative stress.

### 3.3. Transcriptional Nexus of Gibberellic Acid-Induced Antioxidant Pathway Genes in Salinity-Stressed Safflower Seedlings

Under stress, plant redox homeostasis is maintained primarily by a complex antioxidant defense system composed of enzymatic and non-enzymatic components that efficiently scavenge ROS [74]. In the enzymatic antioxidant cascade, SOD, CAT, POD, APX, GPX, GR, DHAR, and GSTs are particularly crucial, acting in series to detoxify superoxide radicals and hydrogen peroxide [74]. Recent research on Arabidopsis highlighted the interplay of GA signaling and stress tolerance, wherein GA influenced the activity and expression of several key antioxidant enzymes critical for managing oxidative stress [75]. In the same way, the interaction between GA and ABA was found to promote GA synthesis and upregulate antioxidant genes, providing insight into how plants enhance their stress resilience at the molecular level [76]. In line with this fundamental antioxidant-related gene expression, our qRT-PCR results also demonstrate that GA_3_ substantially amplifies the transcriptional response of safflower antioxidant-related genes under NaCl stress. The combined GA_3_ + salt treatment significantly upregulated a broad suite of antioxidant genes, including *SOD*, *POD*, *CAT*, *APX*, *GPX*, *GST*, *GR*, and *DHAR* (Figure 4), consistent with the enhanced activity of their corresponding enzymes (Figure 3).

Noticeably, we also observed that GA_3_ alone induced multiple antioxidant genes, whereas NaCl stress alone triggered only limited or inconsistent transcriptional activation (Figure 4). This interrelationship between antioxidant and GA_3_ signaling provides compelling evidence of how the transgenic expression of antioxidant enzymes results in reduced ROS levels, consequently enhancing drought and NaCl tolerance in various plant species [77,78]. Additionally, studies in *Pisum sativum* have detailed the role of antioxidant defense systems in responding to salt stress. A previous study revealed that increased activation of SOD, CAT, and APX was directly correlated with GA application, which plays a vital role in repairing oxidative damage and restoring cellular function [79]. Another finding on the halophyte *Aeluropus littoralis* further demonstrates that GA treatments significantly promote the activity of antioxidant enzymes under prolonged salinity stress, adding to the body of evidence that climate resilience in plants often hinges on GA-mediated antioxidant expression [80]. This strong congruence between expression pattern and the enzymatic activity of the antioxidant nexus in safflower seedlings indicates a sophisticated regulatory mechanism by which GA_3_ enhances salinity tolerance at both molecular and biochemical levels.

### 3.4. Reciprocal Regulation of Gibberellic Acid-Mediated HSYA Pathway Genes in Safflower Seedlings Exposed to Salt Stress

Secondary metabolites such as flavonoids play essential roles in mitigating oxidative stress by scavenging ROS and stabilizing cellular redox balance [38,44]. Their ability to prevent ROS formation is mediated through three primary mechanisms: the inhibition of ROS-generating enzymes, the recycling of other antioxidants, and the chelation of transition metal ions [81]. However, growing evidence reveals that abiotic stresses compromise the natural antioxidant capacity of plants by downregulating key flavonoid pathway genes [82,83,84,85]. In this study, we demonstrate that exogenous GA_3_ enhances the accumulation of both HSYA and quercetin, while counteracting salt-induced suppression of their biosynthetic pathways. GA_3_ application in safflower significantly upregulated CtF6H, CtCGT, and Ct2OGD1, enzymes directly involved in the HSYA pathway. Previous reports highlight that hydroxylases and glycosyltransferases are often rate-limiting steps in the synthesis of safflower-specific quinochalcone glycosides [86,87]; therefore, the strong transcriptional response of these genes to GA_3_ suggests that GA signaling preferentially enhances the HSYA branch of the pathway. The significant increase in HSYA content under GA_3_ alone and combined GA_3_ + NaCl treatments further supports the functional link between GA_3_-induced transcriptional activation of flavonoid biosynthesis.

Salt stress alone led to a substantial decline in both HSYA and quercetin levels, which aligns with earlier findings in safflower and other oilseed crops reporting that salinity represses early phenylpropanoid enzymes such as CHS and CHI, thereby limiting flavonoid biosynthesis [85,88]. In this study, NaCl caused strong downregulation of *CtF6H*, *CtCHI1*, and *CtCGT*, confirming that the pathway is highly sensitive to ionic and osmotic stress. Similar inhibitory effects on *CHS*/*CHI* expression under NaCl have been reported in grapes [89] and tomato [90], where reduced transcription led to decreased flavanol accumulation and heightened oxidative damage. Most importantly, a notable finding of this study was the partial-to-full restoration of HSYA pathway gene expression when GA_3_ was combined with salt stress. This recovery effect is consistent with previous reports in *Pisum sativum* [41], wheat [91], and rice [92], showing that GA_3_ mitigates salt-induced repression of secondary metabolic pathways by rebalancing hormonal homeostasis and maintaining carbon flux toward protective metabolites. The improved HSYA accumulation under GA_3_ + NaCl indicates that HSYA is more responsive to GA_3_-mediated regulation than quercetin, likely due to the strong activation of *CtF6H*, *CtCGT*, and *Ct2OGD1* genes expression. Studies have shown that HSYA is a well-known key antioxidant metabolite identified in safflower, which helps in protection against oxidative stress [92].

**Figure 6:**
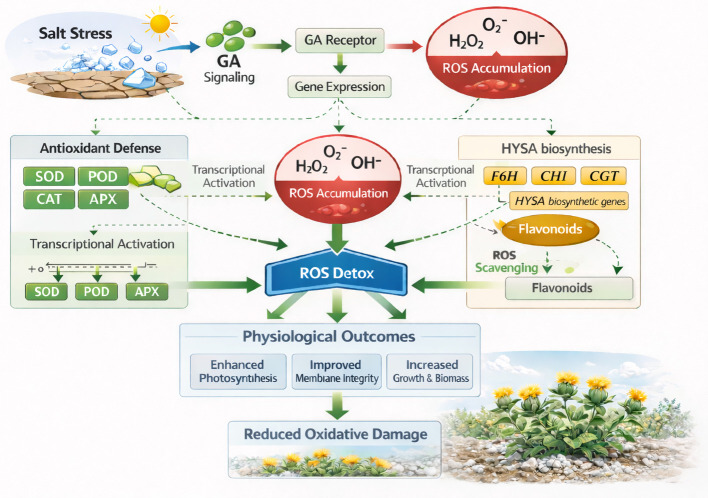
Proposed model illustrating GA_3_-induced antioxidant-HSYA regulation complexes to alleviate salinity stress in safflower. Exogenous application of GA_3_ triggers compensatory responses that mitigate salinity-induced damage by activating two major regulatory networks: (i) the antioxidant gene expression network, including key ROS-scavenging genes such as SOD, POD, CAT, GPX, and APX; their upregulation enhances enzymatic activity involved in detoxifying ROS, thereby stabilizing antioxidant capacity. (ii) The flavonoid/HSYA-related biosynthetic gene network, involving C4H, CHI, F6H, CGT, and OGD; GA_3_ stimulation promotes the transcriptional activation of these genes, leading to a higher metabolic accumulation of HSYA. Enhanced antioxidant enzyme activity and increased HSYA biosynthesis synergistically contribute to improved oxidative balance and overall stress tolerance.

The quercetin biosynthesis pathway exhibited a notably complex and differential regulatory response to NaCl stress. While salt stress significantly induced the expression of *CtCHS*, the downstream *flavanone 3-hydroxylase* (*CtF3H*) remained persistently repressed across all treatments (Figure 5D). This imbalance matches the previous findings in Arabidopsis [93] and safflower flavonoid studies [94], where *F3H* is considered a key regulatory bottleneck under abiotic stress. In safflower, this indicates that, while GA_3_ positively influences the upstream and mid-branch steps of the quercetin pathway, it does not effectively overcome the downstream block presented by *CtF3H*, suggesting that *F3H* acts as a regulatory point that could limit flavonoid accumulation under stress conditions. The persistent suppression of *CtF3H* aligns with recent reports highlighting the pivotal role of *F3H* in the flavonoid biosynthesis pathway [93]. Despite the strong activation of *CtCHS*, which contributes to initial flavonoid synthesis, the lack of subsequent increases in quercetin biosynthesis implies that GA_3_ influence may not sufficiently counteract the bottleneck effects at later stages of pathway regulation. The unchanged expression of *dihydroflavonol 4-reductase* (*CtDFR*) across treatments further suggests that GA_3_ effects are not extended to downstream reductive processes in quercetin biosynthesis. The most substantial increase in quercetin content observed under GA_3_ + NaCl treatment suggests that GA_3_ partially mitigates the negative impact of NaCl on quercetin levels, although not as efficiently as in the HSYA pathway. From these findings, we imply that GA_3_ acts as a positive regulator of flavonoid biosynthesis under NaCl stress, with a particularly strong influence on the HSYA pathway (Figure 6). By reversing the salt-induced suppression of key biosynthetic genes and sustaining the accumulation of antioxidant flavonoids, GA_3_ enhances safflower’s capacity to mitigate oxidative damage. These findings support the broader view that hormonal, metabolic integration is an essential strategy for maintaining redox stability under salinity and highlight GA_3_ as a promising tool for improving stress tolerance in safflower. Overall, these results reinforce the rationale that GA_3_ functions as a positive regulator of flavonoid biosynthesis under conditions of salinity stress. By reversing the suppression of key biosynthetic genes and supporting the accumulation of antioxidants and flavonoids, GA_3_ is potentially involved in safflower responses against oxidative damage. This highlights the essential role of hormonal and metabolic integration in sustaining redox balance, thereby highlighting the essential role of GA_3_ as a potent stress-tolerant growth hormone in safflower.

## 4. Conclusions

The present study demonstrates that exogenous GA_3_ application effectively mitigated the adverse effects of salinity stress on safflower growth and metabolism. The use of GA_3_ at an optimized concentration of 400 mg L^−1^ reactivated key components of the antioxidant–flavonoid network under salinity stress, leading to enhanced activity and coordinated transcriptional induction of major antioxidant enzymes, ensuring effective ROS detoxification. In parallel, metabolite and gene expression analyses also revealed GA_3_-mediated stimulation of the flavonoid biosynthetic pathway, particularly the HSYA branch. This GA_3_-driven redox–metabolic coordination provides clear mechanistic insight into the hormonal regulation of salt tolerance and offers a practical strategy for improving the stability, nutritional quality, and functional value of safflower under saline conditions.

## 5. Materials and Methods

### 5.1. Experimental Design of Plant Materials and Growth Conditions

The safflower cultivar “Jihong No. 1” was used as the experimental material in this study. The experiment was conducted in May 2024 under controlled environmental (artificial climate chamber) and field conditions at the Bioreactor and Drug Development Engineering Research Center of Jilin Agricultural University. Safflower seeds were prepared by performing brief vernalization treatment via dark soaking at 10 °C for 16 h for germination. Afterwards, the germinated seedlings were maintained in the climate chamber; the plants were at a constant 25 °C under a 16 h photoperiod with 80% ± 5% relative humidity and a light intensity of 350 µmol m^–2^ s^–1^ to ensure optimal growth. The soil growth substrate consisted of humus soil, vermiculite (8:2, *v*/*v*) that was uniformly mixed and autoclaved at 121 °C for 30 min. The experimental setup was designed with a sowing density of 5 seeds per meter and experimental plots followed a strip-sowing design (8 m width × 50 m length, 1 m row spacing), excluding 1 m boundary zones at both ends. No fertilizers were applied during sowing and weeds were manually controlled.

### 5.2. Screening Optimal GA_3_ Concentration and Induction of Salt Stress

The first experimental phase was conducted under non-stress conditions to optimize GA_3_ dosage and identify the responsive developmental stages of safflower. For this purpose, uniform and healthy safflower plants were selected and subjected to six GA_3_ treatment concentrations: 0 (distilled water control) (1), 100 (2), 200 (3), 300 (4), 400 (5), and 500 mg L^−1^ (6). GA_3_ concentration was the main influence factor and the tissue/growth stage (seedling, stem, and flower bud stages) was the secondary influence factor. The GA_3_ solutions were freshly prepared and applied as a foliar spray until runoff using a hand-held sprayer. Treatments were administered once every 3 days for three consecutive applications at each stage; control plants received the same volume of distilled water. The flower bud stage was chosen when floral buds (0.5 cm diameter) developed at shoot apices.

The second experimental phase was conducted using the optimized GA_3_ dose (400 mg L^−1^) to evaluate GA_3_-mediated mitigation of salinity stress. Briefly, the safflower plants with fully grown stem tissue were further divided into four groups, including (1) CK (distilled water), (2) GA_3_ alone (400 ppm), (3) NaCl stress (100 mM), and (4) combined GA_3_ + NaCl stress (150 mM NaCl + 400 ppm GA_3_). During the combined GA_3_ + NaCl treatment, NaCl was applied 8 h before GA_3_ treatment. An NaCl concentration of 100 mM was selected on the basis of previous studies which suggest this concentration as a single, clear “moderate-to-severe” stress level (reported for seedlings, callus assays, and vegetative screening in safflower) [94]. Similarly, the 8-hour interval was selected to allow sufficient initiation of salt-stress signaling and early oxidative responses before GA_3_ application, thereby enabling assessment of GA_3_ as a mitigating rather than preventive treatment. All treatments were applied using Hoagland solution in soil medium in growth chambers marked suitable for safflower [95]. Three biological replicates were established for each treatment group. The samples were collected on the tenth day and were stored at −80 °C.

### 5.3. Phenotypic Evaluation Under Optimal GA_3_ Application and Combined GA_3_-Salinity Treatments

Firstly, phenotypic screening was performed to determine the optimal GA_3_ concentration for safflower growth promotion using different concentrations at three developmental stages, seedling, stem elongation, and flower bud initiation. Visual observations included shoot elongation, leaf size, and floral initiation. Quantitative traits measured included leaf biomass (fresh weight expressed in g) and plant height (cm), which were recorded immediately after GA_3_ application using a digital ruler. Photosynthetic parameters were also measured to evaluate the physiological effects of GA_3_ concentration gradients. Photosynthetic rate (Pn) and transpiration rate (Tr) were recorded using a portable photosynthesis system (LI-6400XT; LI-COR Biosciences, Lincoln, NE, USA). Measurements were conducted on the fully expanded uppermost functional leaf of each plant under the following standardized conditions: photosynthetically active radiation (PAR): 1000 μmol m^−2^ s^−1^, CO_2_ concentration: 400 μmol mol^−1^, leaf chamber temperature: 25 ± 1 °C, relative humidity: 50–60%, and airflow rate: 500 μmol s^−1^. The plants were acclimated in the chamber for 2–3 min before data collection. Three independent plants per treatment were used and readings were averaged from three stable measurements per leaf.

Secondly, the phenotypic evaluation of the optimal GA_3_ concentration (400 ppm) together with the NaCl stress (100 mM), both individually and in combination, was conducted. The visible stress symptoms and growth performance, including shoot height, leaf area, chlorosis and necrosis severity, leaf rolling/curling, plant vigor, and overall canopy morphology, were analyzed. Photographs were taken under consistent lighting and angles for comparative analysis.

### 5.4. Determination of MDA and Proline Content

Approximately 0.1 g of plant samples from each treatment (control, GA_3_, NaCl, and GA_3_ + NaCl) was homogenized in 1 mL of 5% (*w*/*v*) trichloroacetic acid (TCA). The homogenate was thoroughly ground to obtain a uniform slurry and MDA quantification was used. Briefly, the mixture was centrifuged at 4000 r/min for 10 min to obtain a clear extract. Then, 500 μL of the extract was mixed with 500 μL of 0.6% (*w*/*v*) thiobarbituric acid (TBA) solution, thoroughly vortexed, and heated in a boiling water bath for 10 min. After cooling to room temperature, the reaction mixture was centrifuged again at 3000 r/min for 15 min. The absorbance of the resulting supernatant was recorded at 532, 600, and 450 nm (A_532_, A_600_, and A_450_). The MDA content (nmol g^−1^) was calculated using the following formula:

MDA (μmol g^−1^) = C × V^T^ × V_1_/(1000 × V_2_ × W)

where V^T^ is the total volume of the extract, V_1_ is the volume of extract mixed with TBA, V_2_ is the volume of extract used for reaction, and W is the weight of the sample.

For proline content, an equal amount of 0.1 g of plant samples under the same treatment conditions as discussed above was homogenized in 150 μL of 80% (*v*/*v*) ethanol. The combined extract was transferred to a test tube and the final volume was adjusted to 10 mL with 80% (*v*/*v*) ethanol. The samples were extracted at room temperature in the dark for 24 h. After extraction, the mixture was filtered and centrifuged and the clear supernatant was retained for analysis. For the colorimetric reaction, 100 μL of the extract was mixed with 100 μL of glacial acetic acid and 100 μL of ninhydrin reagent, sealed, and heated in boiling water for 15 min. After cooling, 250 μL of toluene was added, mixed thoroughly, and allowed to stand in the dark until phase separation occurred. The upper toluene layer was collected and absorbance was measured at 520 nm (A_520_).

Proline content (μg g^−1^) was calculated using the following formula:

Proline = (C × V_T_)/(W × V_S_)

where C is the amount of proline obtained from the standard curve, V_T_ is the total extract volume, V_S_ is the volume of extract used for the reaction, and W is the fresh weight of the sample. The methodology was adopted from the protocol described by [96].

### 5.5. Determination of ROS Content

To determine H_2_O_2_ content, 2 g of fresh plant sample was homogenized with an equal volume (1:1, *w*/*v*) of cold acetone using a chilled mortar and pestle. The homogenate was transferred to a centrifuge tube and spun at 3000 rpm for 10 min at 4 °C. Then, 1 mL of the previous extract was mixed with 0.1 mL of titanium sulfate solution and 0.2 mL of concentrated ammonia solution until precipitation. This mixture was centrifuged again at 3000 rpm for 10 min and washed 3–5 times with cold acetone to remove pigments. The cleaned precipitate was then dissolved in 2 mol/L sulfuric acid and the absorbance was measured at 415 nm using a spectrophotometer. The H_2_O_2_ concentration was determined from a standard curve and expressed as μmol per gram of fresh weight using the following formula:

H_2_O_2_ content (μmol/g FW) = (C × VT)/(W × VS),

where C is the concentration from the standard curve, VT is the total volume of extract, VS is the volume used for the assay, and W is the fresh weight of the sample. The method was adopted from [97] with minor modifications.

For the measurement of superoxide radical $0_{2}^{-}$ content, an equal amount (0.1 g) of fresh plant material under the same treatment conditions as discussed above was homogenized in 200 μL of 50 mmol/L phosphate-buffered saline (PBS, pH 7.8). The homogenate was centrifuged at 5000 rpm for 10 min and the resulting supernatant was collected. Then, a 100 μL aliquot of the supernatant was mixed with 90 μL of 50 mmol/L PBS and 10 μL of hydroxylamine hydrochloride and then incubated in a water bath at 25 °C for 30 min. After incubation, 200 μL of the reaction mixture was combined with 1 mL of p-aminobenzenesulfonic acid and 200 μL of α-naphthylamine, followed by a second incubation at 25 °C for 20 min. Finally, 600 μL of n-butanol was added to the reaction mixture, which was thoroughly mixed, and the absorbance was measured at 530 nm (A530) using a spectrophotometer. The method was adopted from [98] with slight changes.

### 5.6. Determination of Total Flavonoid Content

Exactly 0.1 g of fresh plant sample from each treatment was minced into a fine powder in liquid nitrogen. The powdered sample was immediately transferred to a 10 mL centrifuge tube, followed by the addition of 0.5 mL methanol. Subsequently, 0.15 mL of 5% (*v*/*v*) sodium nitrite solution was added and the mixture was allowed to stand for 6 min. Then, 2 mL of 4% (*v*/*v*) sodium hydroxide solution was added, after which 0.15 mL of 10% aluminum nitrate solution was added and the mixture was left to stand for another 6 min. The volume was adjusted to 5 mL with distilled water and the mixture was thoroughly shaken and allowed to stand for 3 min. Finally, the sample was centrifuged at 10,000 rpm for 10 min, and 0.3 mL of the supernatant was transferred to a spectrophotometric plate for analysis at 508 nm. The above method was adopted from [99] with slight modifications.

### 5.7. Determination of Soluble Sugar and Protein Content

Fresh plant samples (1.0 g) from each treatment (control, GA_3_, NaCl, and GA_3_ + NaCl) were placed into a test tube. After adding 15 mL of distilled water, the mixture was boiled for 20 min, cooled, and filtered into a 100 mL volumetric flask. The residue was washed with distilled water and the volume was adjusted to 100 mL. An aliquot of 1.0 mL of the extract was mixed with 5 mL anthrone reagent, boiled for 10 min, cooled to room temperature, and the absorbance was recorded at 620 nm. Soluble sugar content was calculated using the following formula:

Soluble sugar (%) = (C × VT)/(10^6^ × W × V_1_) × 100,

where C is glucose content from the standard curve, VT is total extract volume, V_1_ is the extract volume used for the reaction, and W is the sample weight. The above method was adopted from [100] with slight modifications.

For soluble protein content, approximately 0.5 g of fresh plant material from each group was homogenized with 5 mL distilled water and centrifuged at 3000 r/min for 10 min. The supernatant was collected and 1.0 mL was mixed with 5 mL Coomassie Brilliant Blue reagent. After standing for 2 min, the absorbance was measured at 595 nm. Protein content was calculated according to the following formula:

Protein content = (C × VT)/(1000 × WF × VS),

where C is the value obtained from the standard curve, VT is the total extract volume, WF is the fresh weight of the sample, and VS is the extract volume used for measurement. The above method was adopted from [101] with slight modifications.

### 5.8. Determination of Chlorophyll Content and Antioxidant Enzyme Activity

Fresh safflower samples were washed, chopped, and 0.9 g of tissue was weighed and divided into three portions (0.3 g each). Each portion was placed in a mortar with quartz sand and calcium carbonate, followed by the addition of 2–3 mL of 95% ethanol. The samples were ground into a paste and an additional 10 mL of ethanol was added until the tissue turned white. The homogenate was allowed to stand for 3–5 min. Then, the extract was filtered through ethanol-moistened filter paper into a 25 mL brown volumetric flask. The filter residue was washed with ethanol until colorless and the volume was brought to 25 mL and the extract was transferred to a cuvette, with 95% ethanol as the blank. The content of chlorophyll a (Ca), chlorophyll b (Cb), and total carotenoids (Cx) were determined spectrophotometrically [102] using absorbance at 665, 649, and 470 nm, respectively, following standard equations.

Ca = 13.95A_665_ − 6.88A_649_  Cb = 24.96A_649_ − 7.32A_665_        Cx = (1000A_470_ − 2.05Ca − 114Cb)/245

Total pigment content was expressed as:

Chlorophyll content = pigment concentration × extract volume × dilution factor/fresh sample weight.

The quantification of and enzymatic activity of key antioxidant indicators such as SOD, POD, CAT, GR, APX, GPX, GST, Prx, and DHAR were investigated from the plant samples obtained after different treatments using different reagent kits purchased form Suzhou Comin Biotech Co., Ltd. (Suzhou, China). The method was adopted from [95] with minor modifications.

### 5.9. Expression Profiling Using Real-Time Quantitative PCR

The total RNA was extracted from safflower plants under the different treatments described above, using RNAiso Plus (Takara, Dalian, China; Cat. No. 9108Q) and following the manufacturer’s instructions. The extracted RNA was then reverse-transcribed into cDNA using the PrimeScript™ RT Reagent Kit with gDNA Eraser (Takara, Dalian, China; Cat. No. RR047Q) to ensure the removal of genomic DNA contamination. Real-time quantitative PCR (qRT-PCR) was performed using MonAmp™ ChemoHS Specificity Plus qPCR Mix (Low ROX) (Monad, Suzhou, China; Cat. No. MQ00601S) on a real-time PCR system. The 18S ribosomal gene was used as the internal reference gene to normalize gene expression levels after confirming Cq stability (SD < 0.5). The relative expression levels of the target genes were calculated using the 2^−ΔΔCT^ method [103], where ΔΔCt = (Ct_target gene-Ct_reference gene)-(Ct_control gene- Ct_reference gene). Each experiment included three biological replicates and three technical replicates to ensure reproducibility and accuracy. The gene-specific primers used in this study are listed in Supplementary Table S1.

### 5.10. Quantification of Quercetin and HSYA Content Using HPLC

An Agilent XDB-C18 column (250 mm × 4.6 mm, 5 μm) was selected for the liquid chromatography analysis of both quercetin and HSYA. For quercetin, the mobile phase consisted of 0.4% formic acid (A) and methanol (B) in a ratio of 5:5. The detection wavelength was set at 255 nm, the flow rate at 1 mL/min, and the column temperature at 25 °C. These conditions were optimized through multiple experiments to ensure the effective separation and detection of quercetin. The content of quercetin was calculated using the following formula:

Content (mg/g) = C_control*A_sample*V_constant/A_control*M_sample.

For hydroxysafflor yellow A, the mobile phase was also 0.4% formic acid (A)-methanol (B), but with a ratio of 7:3. The detection wavelength was 402 nm, and the flow rate and column temperature were the same as those for quercetin detection, i.e., 1 mL/min and 25 °C. The content of hydroxysafflor yellow A was calculated using the same formula:

Content (mg/g) = C_control*A_sample*V_constant/A_control*M_sample.

### 5.11. Statistical Analysis

All experiments were conducted using a completely randomized design with at least three biological replicates per treatment. Each biological replicate consisted of pooled tissues from three independent plants, and three biological replicates were used per treatment. For biochemical assays and qRT-PCR analyses, three technical replicates were performed for each biological replicate. Data are presented as mean ± standard deviation (SD). Prior to statistical analysis, datasets were tested for normality using the Shapiro–Wilk test and for the homogeneity of variances using Levene’s test. Mean comparisons were performed using one-way analysis of variance (ANOVA). Post hoc multiple-comparison tests were applied to determine statistically significant differences among the treatment means. Statistical significance is indicated by asterisks: * *p* ≤ 0.05, ** *p* ≤ 0.01 and *** *p* ≤ 0.001.

## Data Availability

The original contributions presented in this study are included in the article/Supplementary Material. Further inquiries can be directed to the corresponding author.

## Supplementary Materials

The following supporting information can be downloaded at: https://www.mdpi.com/article/10.3390/plants15020267/s1, Figure S1: The experimental setup, pot arrangements and growth environment of growing safflower JH1 cultivar used in this research. Table S1: List of Primers used in this study.
