# Supplementary material for: Gibberellic Acid-Induced Regulation of Antioxidant–Flavonoid Channels Provides Protection Against Oxidative Damage in Safflower Under Salinity Stress
Source: Plants (Basel). 2026 Jan 15;15(2):267. doi: 10.3390/plants15020267 (PMC12844619; doi:10.3390/plants15020267)
Supplement: Supplementary file 1 [file plants-15-00267-s001.zip › plants-4079638-supplementary.pdf]

Figure S1

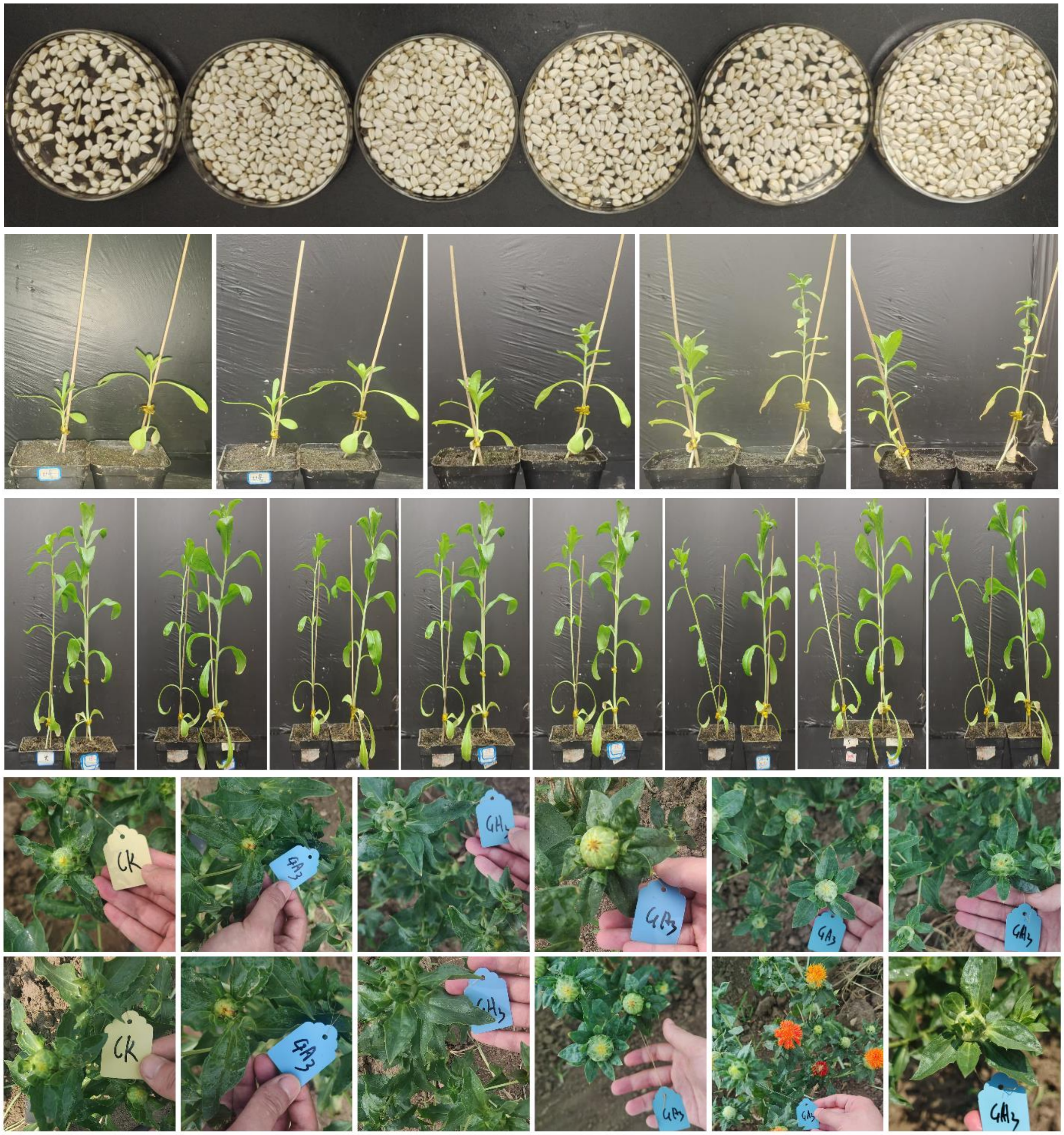

Figure S1: The experimental setup, pot arrangements and growth environment of growing safflower JH1 cultivar used in this research.

Table S1:

List of Primers used in this study.

Gene	Accession No.	Forward Primer (5′–3′)	Reverse Primer (5′–3′)	product (bp)	Tm
CtF6H	PQ040214.1	TGAGAAGGCTGGTGCTGAAG	CAGCAGCTTCTTCTTCAGCG	135	60
CtCGT	PQ040212.1	AGGGTTCAGGCTTCTTCGAC	TGTAGCCACAGCAGGACTTG	142	60
Ct2OGD1	PQ040215.1	ACCTTCGAGCAGCCTTCAAC	TTGATGTCCAGGAGGTGCTG	128	60
CtCHI1	PV014870.1	CGACTTCGAGGTGCTGGTTA	ACGATGACCGTGACCTTGAA	131	60
CtC4H	JN998608.1	TCTGCTCTTCGAGGCTTCTC	GAGATGACCAGCCAGTGTCA	128	60.1
CtCHS	MG759483.1	AGGACTTCTGGTGACCACCA	CCTTGAGGCTGTAGGAAGGA	140	59.8
CtF3H	JF737995.1	CCAGAGGAGGGAAGAGGAAG	TGGTGAGATGGTGAGGTGGT	132	60.3
CtDFR	OP616395.1	GCCTGCTTCTTCTTGCTTCC	TCGTCCTTGGTCTTGGTGTT	138	60.0
CtPOD1	LC634056.1	TGGCTGCTTCTCTGCTTCAT	GGTGGTAGTGGTTGGTGATG	142	60.0
CtPOD2	LC634057.1	CCTGCTGGTGTTGTTGTTGA	AGGACCATGGTCAAGATGGC	136	59.7
CtPOD3	LC634058.1	CGAGGTTGTCGATGAAATCA	CGTGTTCGATAATCAGTATTTCA	148	60.2
CtPOD4	LC644555.1	TGGAGCCTCTTAAGGAGCAG	CCTCATATCTTTGACAACAGCTAC	129	59.9
CtPOD5	LC644556.1	TCTTGATGATACACCTTCTTTCATC	GGTTGATATCCAATCTCCGACC	151	60.1
CtCAT1	CCG012179.1	GCTGCTGGTGTTGTTGTTGA	CGTCCAGGAGTCCAGACCTA	138	60.1
CtCAT2	CCG028606.1	GTTGCTGCTGATTTTCTTCG	CCTGGTCCAGGTTCAGATGT	145	59.8
CtAPX1	CCG019778.1	GCTGGTGTTGATGATGCTGA	CGAGGTAGTTGAGGCTGTTG	136	60.0
CtAPX2	CCG007556.1	CAGTTTGATAATTCCTACTTCAAGGT	GCTAGTTGTTGCTGTTGAATTC	142	59.6
CtGPX1	CCG006096.1	GCTAGTTCTCTTCCATATATGTTGG	CCTTGTTGAACAAAGTAATAAGAAAA	138	59.8
CtGPX2	CCG006097.1	GCTGTGTTTGGCAAAAATAAGCTA	GGTTCGTTGCACCAACAAGC	145	60.1
CtGPX3	CCG013943.1	GGTTAATGTTGCATCCAAATGGTAC	CAGTTTGGTCTTTTAACCAAGTGG	132	59.6
CtGR	CCG002820.3	GCTGGTAGCAGGGCGGCACG	CTTGATGCTTCTAGATGAATTTGG	138	60.2
CtDHAR1	CCG011398.1	GCTAGATGAAGGGCCAAGAAC	CTTGGTGATGATTTTTTACTTTAGTT	132	59.8
CtDHAR2	CCG000521.1	GGTGAACTCTGCATCATCTCCG	CCAAATTCATTATTTAATGTTGTATATT	141	60.1
CtGST1	CCG008533.1	CGTGGTTCAATTAAAATTAAAAACAA	CTTGGTGGTCACTTTGGAGGT	138	59.1
CtGST2	CCG026530.1	GACGACGATTTTATACCCGAAAC	GTGACGCTTCGTGAACAAGAAG	142	58.7
CtGST3	CCG026541.1	GGTTTTGGAAAGAAAGCGAACA	CGCTGATATAATTGTTTTTTCCACT	131	58.9
18srRNA	-	TTATCCTAGTTTGCGCGCTA	GAATCCTGTTGCCGGTCTTG	145	60
